# Using Information from the Electronic Health Record to Improve Measurement of Unemployment in Service Members and Veterans with mTBI and Post-Deployment Stress

**DOI:** 10.1371/journal.pone.0115873

**Published:** 2014-12-26

**Authors:** Christina Dillahunt-Aspillaga, Dezon Finch, Jill Massengale, Tracy Kretzmer, Stephen L. Luther, James A. McCart

**Affiliations:** 1 Center of Innovation on Disability & Rehabilitation Research (CINDRR) James A. Haley Veterans Hospital, Tampa, Florida, United States of America; 2 Department of Rehabilitation and Mental Health Counseling, University of South Florida, Tampa, Florida, United States of America; 3 James A. Haley Veterans Hospital, Tampa, Florida, United States of America; University of Waterloo, Canada

## Abstract

**Objective:**

The purpose of this pilot study is 1) to develop an annotation schema and a training set of annotated notes to support the future development of a natural language processing (NLP) system to automatically extract employment information, and 2) to determine if information about employment status, goals and work-related challenges reported by service members and Veterans with mild traumatic brain injury (mTBI) and post-deployment stress can be identified in the Electronic Health Record (EHR).

**Design:**

Retrospective cohort study using data from selected progress notes stored in the EHR.

**Setting:**

Post-deployment Rehabilitation and Evaluation Program (PREP), an in-patient rehabilitation program for Veterans with TBI at the James A. Haley Veterans' Hospital in Tampa, Florida.

**Participants:**

Service members and Veterans with TBI who participated in the PREP program (N = 60).

**Main Outcome Measures:**

Documentation of employment status, goals, and work-related challenges reported by service members and recorded in the EHR.

**Results:**

Two hundred notes were examined and unique vocational information was found indicating a variety of self-reported employment challenges. Current employment status and future vocational goals along with information about cognitive, physical, and behavioral symptoms that may affect return-to-work were extracted from the EHR. The annotation schema developed for this study provides an excellent tool upon which NLP studies can be developed.

**Conclusions:**

Information related to employment status and vocational history is stored in text notes in the EHR system. Information stored in text does not lend itself to easy extraction or summarization for research and rehabilitation planning purposes. Development of NLP systems to automatically extract text-based employment information provides data that may improve the understanding and measurement of employment in this important cohort.

## Introduction

Traumatic Brain Injury (TBI) is referred to as the “signature injury” of modern warfare due to the types of explosives used and the improved survivability of those injured in combat [1], [2]. TBI is defined as “a traumatically induced structural injury and/or physiological disruption of brain function as a result of an external force that is indicated by new onset or worsening of at least one of the following signs or symptoms immediately following the event: (a) any loss of consciousness (LOC); (b) any loss of memory for events immediately before or after the event; (c) any alterations in mental state; (d) neurological deficits that may or may not be transient; and (e) intracranial lesion” [3]. TBI severity is categorized as mild, moderate or severe based on the length of LOC, alteration of consciousness or post-traumatic amnesia (PTA) at the time of injury and not by the severity of symptoms at various points in time after injury [3], [4]. Researchers have a consensus about the definition of moderate to severe TBI, but the definition of mild TBI (mTBI) has been elusive [5], [6]. The VA/DOD adopted the American Congress of Rehabilitation Medicine (ACRM) definition of mTBI that specifies that mTBI does not exceed PTA for greater than 24 hours, Glasgow Coma Scale (GCS) of 13 to 15 after the first 30 minutes; and/or LOC for more than 30 minutes and absence of traumatic lesion when radiographic studies are obtained [3], [4], [7].

Employment has been emphasized as a key component of community reintegration for persons with disabilities [59], [60]. Return to work after sustaining a TBI is a common goal across civilian and veteran populations. Due to the consequential nature of TBI, returning to work after injury is often a challenge [20], [26], [27], [29], [38], [57]. Failure to return to work can lead to poor psychosocial outcomes such as decreased community integration and decreased financial independence [27], [54], [55], [57]. These factors are of particular importance to Veterans with mTBI when they return from duty and attempt to reintegrate into civilian life. Veterans may not be diagnosed with mTBI until they experience difficulties reintegrating into society [22], [58]. The cognitive difficulties resulting from the mTBI such as memory loss, attention deficits, and executive control dysfunctions can affect reintegration and successful employment efforts [20], [22], [26], [27], [29], [32], [35], [57], [61], [62].

In this article, we begin with a brief review of current literature available on the complex challenges Veterans with mTBIs face when participating in rehabilitation and transitioning into the community. Then, we describe the TBI Post-Deployment Rehabilitation and Evaluation program (PREP) at the James A. Haley hospital in Tampa, Florida. Next, we provide a brief overview of information extraction (IE) methods used in mining of patient electronic medical records. Information found on Veteran employment status and vocational history stored in text notes located within the Electronic Health Record (EHR) is presented. In conclusion, we discuss how the pilot study findings may improve our current understanding of the reintegration and employment needs of Veterans with mTBIs. Finally, we discuss how development of future natural language processing systems (NLP) may support easy summarization of employment status, goals, and work-related barriers located in text in the EHR.

## Review of the Literature

Since 2000 more than 280,000 incident diagnoses of TBI in all military personnel (not just war-related TBI) have been reported, with a majority of these injuries being classified as mild [8], [52]. Determining the exact incidence and prevalence of deployment-related TBI is challenging due to the fact that mild injuries (mTBI) may not require immediate medical attention and it is possible that these injuries may not be documented directly after the event occurs when acute injury characteristics are most accurately assessed [3].

The rate of TBI in military personnel is higher than in the civilian population [3], [9]. It is estimated that approximately 1 in 6 US service members deployed in Operation Iraqi Freedom (OIF), Operation Enduring Freedom (OEF), or Operation New Dawn (OND) has sustained a TBI, with many of these Veterans experiencing multiple TBIs and complex injuries [3], [10]–[14]. Vast majorities of combat TBIs are blast-related and are often associated with polytrauma, which is defined as two or more injuries to physical regions or organ systems, one of which may be life threatening, resulting in physical, cognitive, psychological, or psychosocial impairments and functional disability [3], [10], [15]–[17]. Blast injuries have been identified as one of the main causes of mTBI in service members who have been deployed [22] and a history of mTBI is common among those who served in OIF/OEF conflicts [14], [26], [63], [64].

In general, TBI typically results in short- and long-term physical, cognitive, emotional, and behavioral consequences that negatively impact an individual's ability to live an independent, integrated, and productive life [18], [19]. Previous research highlights the potential for functional impairment following TBI [10], [19]. Most symptoms of mTBI (e.g., somatic, cognitive impairment, and psychological) will resolve during the first few weeks and months post-injury, although some individuals (5–15%) continue to experience symptoms for long periods of time with some Veterans experiencing cognitive difficulties that impede reintegration into society post-combat [3], [10], [20]–[22], [26], [29]. Complex co-morbidities such as chronic pain, irritability, sleep disturbances, and mental health disorders (e.g., depression, anxiety, PTSD) interfere with work and independent living [16], [20], [23]–[30].

Overall, Veterans with TBI may experience higher rates of unemployment than civilians and have more difficulty obtaining employment compared to those in previous conflicts, while they are often in their prime earning years [10], [31]–[33]. Veterans and service members with TBI may experience high unemployment rates in part because these individuals experience multiple problems when returning to work. The number, complexity, and interaction of problems may contribute to the difficulty in maintaining long-term employment [20], [24], [34], [35], [70]. For example, problems may be due to environmental barriers such as access to reliable transportation or the ability to drive in civilian environments [44]–[46], [56], [67]. Other pre-injury factors, such as premorbid work history, employment status, and income have been associated with more successful return to work [35], [37], [57], [65], [68]–[70].

### PREP Program

The Post-deployment Rehabilitation & Evaluation Program (PREP) is a unique in-patient rehabilitation program located at the James A. Haley Veterans' Hospital in Tampa, Florida. This program provides evaluation and treatment of mTBI and post-deployment difficulties including post-deployment stress. In the PREP program, comprehensive evaluations are completed in the in-patient setting rather than extending the evaluation period over a course of several months. The average length of stay is 3–6 weeks, depending upon rehabilitation and mental health goals, although briefer targeted admissions and extended stays are common. Veterans and service members who complete this program undergo specialized evaluations conducted by physiatrists (rehabilitation medicine physicians), physical/vestibular therapists, occupational therapists, speech and language therapists, recreational therapists, vocational rehabilitation counselors, pain specialists, audiologists, vision therapists, social workers, psychiatrists, sleep medicine specialists, neuropsychologists, and psychologists. An interdisciplinary team of clinicians and providers with expertise in mTBI, chronic pain, and post-deployment stress offer integrated and holistic coordinated care across two phases. This includes a one- to three-week comprehensive evaluation to examine physical, cognitive, and mental health symptoms (Phase 1) and an intensive treatment for post-deployment and combat injuries including PTSD and other readjustment issues (Phase 2).

Patients who complete the PREP program report improvements in their cognitive and physical abilities (85% and 78% respectively), emotional functioning (62%), and reductions in headaches, pain, and dizziness [53]. Overall, 43% of program graduates reported improvement in their self-efficacy, which is associated with greater likelihood of meeting the demands of the work environment and improved quality of life [36], [37], [53]. PREP notes were selected for review because they contained rich information on reintegration and employment needs of Veterans and service members with mTBI.

PREP participants interact with a wide variety of clinicians during their stay; therefore providing a rich environment of the kind of information that might be available throughout the Veterans Health Administration (VHA) Electronic Health Record (EHR). The VHA has used an EHR since the 1970s called VistA. This system records important data regarding a Veteran's encounter with each clinician in both structured data tables and text. In the VHA, vast amounts of information are locked away in EHR in the form of progress notes. As of 2013, there were over 2 billion progress notes in the VHA. Many aspects of a patient's rehabilitation trajectory are recorded in progress notes, including diagnosis, rehabilitation strategies, and patient outcomes. These progress notes provide a rich source of rehabilitation-related data, possibly the largest of its kind in the world. Information in these data could provide valuable insights into methods and outcomes of rehabilitation, particularly those related to employment.

### Information Extraction

Information extraction (IE) is defined as the extraction of predefined types of information from text [72]. There are four primary methods available to implement an information extraction system, including natural language processing (NLP), pattern matching, rules, and machine learning. The use of rules and pattern-matching exploits basic patterns over a variety of structures, such as text strings, part-of-speech tags, semantic pairs, and dictionary entries [73]. Patterns are easily recognized by humans and can be expressed directly using special purpose representation languages such as regular expressions [74]. Regular expressions are effective when the structure of the text and the tokens are consistent, but tend to be one-off methods tailored to the extraction task. Hand coding of regular expressions can involve a complex and time consuming development effort that requires a priori knowledge of all possible patterns that represent the concept being sought. Machine learning techniques can be an effective method for automated knowledge acquisition [75]. Features extracted from the text, such as parts of speech and sentence length, are fed into a learning machine to assist in tasks downstream like word sense disambiguation. The primary disadvantage for machine learning used for IE is that it requires a labeled dataset for training. The primary means of performing IE is natural language processing (NLP). NLP research focuses on developing computational models for understanding natural language [76]. Using tools built around ontologies (controlled vocabularies) like SNOMED-CT [77] have enabled researchers to automate the capture of information in clinical narratives.

Mining patient electronic medical records can be useful for detecting patterns in patient care [78], patient treatment habits [79] and their results. Statistical text mining has been used to determine if patients suffer from comorbidities related to obesity [80] and smoking [81], as well as detecting fall-related injuries [82]. Regular expressions have been used to extract blood pressure values from progress notes [83]. NLP has been useful for extracting medical information such as principal diagnosis [81] and medication use [84] from clinical narratives. This has led to a better understanding of the conditions patients face and how to treat them [85]. Manual chart review for annotation has been used extensively and when appropriate rigor is applied, the information extracted is very reliable and is often used as the reference standard to evaluate IE Systems.

## Current Study

In this study, our first objective was to develop an annotation schema and a training set of annotated notes to support the future development of a natural language process (NLP) system to automatically extract employment information reliably from the EHR. The second objective of our study was to determine if information about employment status, goals, and work-related challenges reported by service members and Veterans with mTBI and post-deployment stress are identifiable in selected notes available in the Electronic Health Record (EHR). While the VA EHR contains information about employment, much of it is stored in text notes and is not easily extracted or summarized. Further, there is not a clear understanding of the reintegration challenges faced by Veterans using Veterans Health Administration (VHA) services and there are no available clinical outcomes readily available through VHA administrative databases [3]. The results of this study are intended to provide a foundation for future prospective studies of the measurement of unemployment of service members and Veterans with mTBI.

## Methods

### Ethics Statement

The Institutional Review Boards at the Veterans Health Administration's Office of Research and Development (VHA ORD) and the University of South Florida approved this study protocol and granted waivers of individual consent based on the absence of individually identifying data. Individual patient identifiers from the EHR were replaced with ID numbers. The Department of Veterans Affairs provided a waiver of HIPAA authorization for research conducted in this study.

### Electronic Health Record Data

In order to investigate employment information in the EHR, we used a convenience sample of service members and Veterans with TBI who participated in the PREP program to examine a variety of sociodemographic and clinical factors that may affect return-to-work (RTW) or other productive activity. In the EHR, progress notes are entered directly by clinicians providing service and stored as text-based records in the database. Progress notes are automatically titled within the system based on the service or provider writing the note (e.g., “Nursing Note,” “Psychology Note,” “Social Work Inpatient Note”).

For this study, we reviewed 16 example notes from all 369 unique note titles available for the cohort and chose six note titles that include the targeted information. Veterans' employment information was extracted from text in the EHR from several note titles including the TBI/Polytrauma Rehabilitation/Reintegration Plan of Care notes. Several elements are included in TBI Care Plans including documentation of patient and family goals and Veteran employment status. Due to previous study findings reporting deficiencies in the TBI Care Plans [3], [51], five additional note titles were reviewed in this study including a) Social Work (SW) Psychosocial, b) Physical Medicine and Rehabilitation Service (PM&RS) Vocational Rehab Consultation, c) PM&RS Vocational Rehabilitation Testing, d) Compensated Work Therapy (CWT) Initial Assessment, and e) OIF/OEF SW Psychosocial notes. These note titles were selected because they contain rich employment information. Note titles reviewed and selected are displayed in Table 1. Specifically, information on employment status, work history, educational history, and future paid and unpaid vocational goals were annotated and extracted from all six of the aforementioned text notes. Patient and clinician commentary about readiness and ability to return to work were also extracted and annotated.

**Table 1:** Note Titles Reviewed and Selected.

Note Titles of Notes Reviewed	Note Titles Selected *	
CWT Program Consult Note		
**CWT Initial Assessment**		**X**
CWT Discharge Note		
Mental Health Consult Note		
OIF/OEF Case Manager Telephone F/U		
OIF/OEF Case Manager Note		
PM&RS TBI Consult Notes		
**PM&RS Voc Rehab Consult Note**		**X**
PM&RS Voc Rehab Progress Note		
**PM&RS Voc Rehab Eval/Testing**		**X**
**Social Work(SW)- Psychosocial Assessment**		**X**
SW TBI Follow-up Contact		
**Social Work OIF/OEF Psychosocial**		**X**
SW Family Support Consult		
Social Rehab Group Note		
**TBI Polytrauma Rehabilitation Re-integration Plan (Care Plan)**		**X**

*Selection based upon richness of employment information found within each note title.

### Participants/Sample Selection

The target population for this pilot study was taken from a larger project and included a cohort of service members and Veterans diagnosed with TBI and post-deployment stress that 1) participated in the PREP program between 2009 and 2012 and 2) had at least one TBI Plan of Care note.

### Research Design/Procedure

In this retrospective cohort study, data from our cohort were extracted from the VHA EHR. Structured administrative data were used to describe demographic information on age, marital status, gender, and ethnicity of the cohort. Also, a chart review was performed to extract additional information from our cohort including other demographic information; work history; employment status, goals, and vocational interests; the number of symptoms associated with mTBI and post-deployment stress; and clinical and sociodemographic factors associated with employment status.

### EHR Chart Review

**Figure 1:**
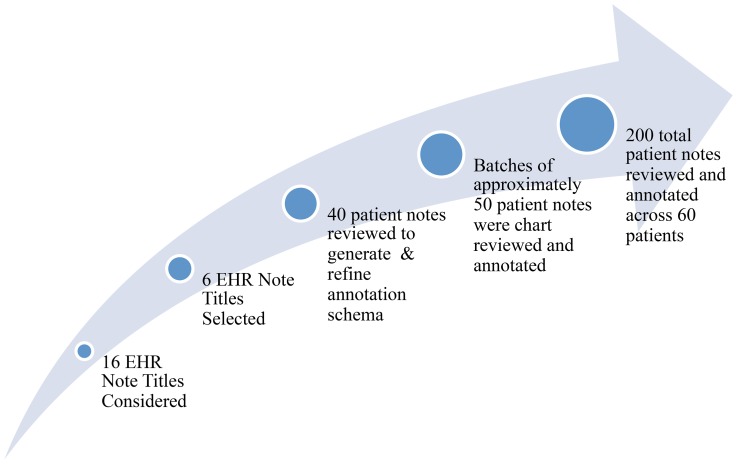
EHR Note Title Selection, Annotation Schema, Chart Review & Annotation.

A chart review was performed on all six aforementioned note titles. In this study, an annotation schema and a training set of annotated notes was generated to support the future development of a natural language processing (NLP) system. Such systems can reliably extract information related to returning to work. Information that can be extracted from the six aforementioned note titles include: vocational status, work history, educational history, driving status, and future paid and unpaid vocational goals The annotation schema used in the chart review was generated and iteratively refined based on insight from a clinical expert and by reviewing an initial batch of approximately 40 notes. Once the annotation schema was finalized, batches of approximately 50 notes were chart reviewed. The process for EHR chart review and annotation is shown in Fig. 1. The annotation software Knowtator [86] was used to chart review all of the notes. Knowtator has a unique advantage over other annotation tools due to its ability to define complex annotation schemas (e.g. schemas which have constrained relationships between entity types) [86].

As part of the chart review, the highest level of education and highest military rank attained were annotated, along with number of unique injuries, location of injury (“in theater” or “stateside”), number of unique deployments, and exposure to blasts (dichotomized as “yes” or “no”). Information about injury severity was collected from the TBI Plan of Care initial note (mild, moderate, or severe) and EHR text information about LOC or PTA was annotated if this information was found (dichotomized as “yes” or “no”). Employment status was classified based on the most current note reviewed. If the service member or Veteran reported being on active duty and unemployed, they were grouped into the active duty category. Reported symptoms were grouped into cognitive, physical, and emotional/behavioral categories as defined at the DOD Force Health Protection and Readiness Conference meeting held in 2007. The presence of specific reports of PTSD, chronic pain, headaches, sensory impairments, and sleep disturbances were also annotated.

### Data Analysis

Data analysis for this project was conducted on the VA Informatics and Computing Infrastructure (VINCI), a secure, virtual computing environment developed through a partnership between the VA Office of Information Technology (OI&T) and the Veterans Health Administration's Office of Research and Development (VHA ORD). VINCI is part of the Transformation for the 21st Century Initiative #13 to provide researchers a nation-wide view of high-value VA patient data. While VINCI brings together data sources and provides the analytical environment for performing studies, VHA National Data Services (NDS) authorizes research access to patient data. New research projects are granted access to snapshots of data that can be updated as needed. In addition to secure data storage, VINCI includes a cluster of servers set aside for tasks like analysis, data processing, and extracting information from text. This allows VA researchers to have access to the data and the applications needed to select, transform, and analyze patient data in a central and secure location accessible from the VA intranet.

## Results

### Descriptive Information for the Sample Population

#### Veteran Demographics

There were 177 Veterans and service members who were enrolled in PREP during the study period (inclusion criterion #1). Of those, 128 Veterans also had a TBI Plan of Care note (inclusion criterion #2). In this pilot study, all of the targeted note titles for 60 Veterans/service members, (n = 200 notes) were reviewed and annotated. Targeted note titles reviewed are displayed in Table 1.

The sample consisted of 60 Veterans and service members (cohort) diagnosed with TBI. Basic demographic data is displayed in Table 2. Most of the cohort in this sample was between the ages of 28 and 32 years (42.0%). The majority of the sample was male (96.7%), White (76.7%), and married (50.0%). In addition, 53.0% of the sample reported completing some college courses but were unable to finish their degree.

**Table 2:** Veterans with TBI Demographic and other Characteristics (N = 60).

Characteristic	N (%)
**Gender**	
Male	58(96.7)
Female	2(3.3)
**Age**	**N (%)**
18–22	1(1.7)
23–27	9(15.0)
28–32	25(41.7)
33–37	8 (13.3)
≥38	17(28.3)
**Race/Ethnicity**	**N (%)**
White/Caucasian	46(76.7)
Black/African American	6(10.0)
Hispanic/Latino(a)	5(8.3)
Native American/American Indian	0 (0.0)
Asian	1(1.7)
Decline	2(3.3)
Not Documented	5(8.3)
**Marital Status**	**N (%)**
Single	15(25.0)
Married	30(50.0)
Divorced	10(16.7)
Widowed	1 (1.7)
Other	4 (6.7)
**Educational Level**	**N (%)**
Less than high school	0
High school diploma/GED	22(36.7)
Vocational training	9(15.0)
Some college*	32(53.3)
Bachelor Degree	5 (8.3)
Graduate/professional degree	1(1.7)
Other^†^	1(1.7)
**Employment Status** ^‡^	**N (%)**
Active Duty	20(33.3)
Employed-Fulltime-Other	1(1.7)
Unemployed	30(50.0)
Part-Time	1(1.7)
Medically Retired	6(10.0)
Other-Student	1(1.7)
Unknown	1(1.7)

Note:* A large percentage of Veterans indicated they took college classes but were unable to finish their degree. ^†^Other  =  Education that was not otherwise specified. ^‡^Status from most recent note reviewed.

#### Veteran and Service Member TBI Injury Characteristics and Symptoms Reported

Many Veterans reported two or more deployments (41.7%), most sustained mild injuries (65.0%), with the most common cause reported being due to a blast (78.3%) in theatre (89.2%). Nearly half of all the Veterans sampled reported LOC but a much smaller proportion reported PTA (55.0% and 13.3% respectively). Injury characteristics and symptoms reported are shown in Table 3 and indicate that there are many commonly reported areas in which cohort members with TBI experience symptoms. Groupings indicate that physical symptoms were reported most frequently (43.9%), followed by emotional/behavioral symptoms (29.8%), and finally cognitive problems (26.3%). Specifically, out of the 60 cohort members, a majority reported PTSD (88.3%), chronic pain (88.3%), headaches (86.67%), and sleep disturbances (76.67%).

**Table 3:** Deployments, Injury Severity, Etiology & Current Symptoms Reported (N = 60).

**Deployment**	**N (%)**
No H/o of deployment	2(3.3)
1 deployment	33(55.0)
≥2 deployments	25(41.7)
Specific Report of Blast Exposure*	**N (%)**
Yes	47(78.3)
No	13(21.7)
**Injury Severity** ^†^	**N (%)**
Mild	39(65.0)
Moderate	4(6.7)
Severe	4(6.7)
NOS	13(21.6)
* LOC Reported* ^‡^	33(55)
* PTA Reported* ^‡^	8(13.3)
**Venue/Location Injury Sustained** ^§^	**N**
In Theatre	74
CONUS/Stateside	9
In training	1
**Cause of Injury** ^‖^	**N**
MVA	12
Blast	47
Fall	17
Pedestrian Injury	0
Assault	8
**Number of Injuries** ^¶^	**N**
Single	84
Multiple	26
unknown	8
**Symptoms Reported** ^#^	**N**
Cognitive	118
Physical	197
Emotional/Behavioral	134
PTSD	53
Chronic Pain	53
Headaches	52
Sensory impairments	62
Sleep Disturbances/Insomnia	46

*Note:* *Veterans may report more than one blast exposure. ^†^Severity as reported in the notes reviewed. ^‡^Specific information found in notes reviewed. ^§^Veterans can report more than one venue of injury. ^‖^Veterans can report multiple and unique causes of injury. ^¶^Veterans can report multiple numbers of injuries. ^#^Symptoms reported at the point in time when case notes were recorded in the EHR record and Veterans can report multiple symptoms.

#### Veteran Military and Employment Descriptions

Specific military and employment information including rank, military occupation specialty (MOS), work history, employment goals, vocational interests, and driving ability is displayed in Table 4. A sizeable portion of the sample had a military rank of E5 (46.7%) and a military occupational specialty of infantry (36.7%). Further, 75.0% of the cohort reported history of civilian work. Many members of the cohort reported future goals of paid employment (65.0%) and returning to school (60.0%). Driving information was found for each member of the cohort. Over half (68.0%) of these individuals were able to drive. Results from the CareerScope assessment profile and associated interest inventory clusters were found in 32% of the sample.

**Table 4:** Military Rank, MOS, Work History, Employment Goals, Vocational Interests & Driving Ability (N = 60).

**Military Rank**	**N (%)**
E3	4(6.7)
E4	28(46.7)
E5	13(21.7)
E6	8(13.3)
E7	3(5.0)
E9	1(1.7)
O3	1(1.7)
O5	1(1.7)
Unknown	1(1.7)
**MOS**	**N (%)**
Infantry	22(36.7)
Mechanical/Trade	13(21.7)
Supply/Logistics	4(6.7)
Medical	6(10.0)
Technical/Communications	7(11.7)
Other	7(11.7)
Unknown	1(1.67)
**Civilian Work History**	**N (%)**
Yes	45(75.0)
Unknown	15(25.0)
Civilian Work History Reported*	**N**
** 0**	15
** 1**	20
** 2**	14
** 3**	8
** 4**	2
** 5**	1
**Future Employment Goals^†^**	**N**
Paid	39
Unpaid/Civic/Volunteer	4
Return to School	36
Other	9
Not ready to set a goal	1
Unknown	2
**Vocational Interests Areas Reported^‡^**	**N (%)**
Yes	19(31.7)
No	41(68.3)
**Driving Ability**	**N (%)**
Able to drive	41(68.3)
Not able to drive - Medical reason	9(15)
Not able to drive - Suspended/revoked license	3(5)
No Car	5(8.3)
Unknown	2(3.3)

*Note*:* Veteran may report more than one previous work history. ^†^Veteran many have more than one future goal. ^‡^Veterans had a record of a CareerScope assessment including interest inventory clusters.

## Discussion

Most individuals with mTBI return to work within a few months post-injury, but those who continue to experience persistent symptoms and functional impairments months' to years' post-injury may encounter limitations in work and other areas of social functioning [20], [21], [29]. Previous research has found that multiple variables need to be considered when studying employment following mTBI in civilian populations as well as military populations [10], [20], [26], [27], [38]. In this pilot study, we explored whether the VHA EHR contains information that could provide insights regarding the levels of unemployment among veterans and service members with mTBI. We confirmed that limited administrative (structured or coded) data in the EHR are available on the employment status of Veterans and service members with mTBI. However, data extracted in this study reveal that information about employment and factors that may affect return-to-work are available in text notes in the EHR. Currently, this information is not readily available for review by clinicians for interdisciplinary rehabilitation planning purposes. Having this information available may assist clinicians concurrently address health-related and vocational needs, which may facilitate successful adjustment and reintegration for Veterans and service members [71].

Through annotation and data extraction, we found that service members and Veterans who participated in the PREP rehabilitation program were mostly Caucasian males between the ages of 28 and 32 who reported being unemployed and noted a variety of symptoms and post-deployment stress. Comparable to many previous research findings [10], [32], [33], [39], we found that many service members and Veterans reported mTBI (65%), PTSD (83%), chronic pain (88%), headaches (87%), sleep disturbances (77%), and unemployment (75%). It is important to note that Veterans and service members in this study completed the in-patient PREP program, which specializes in the evaluation and treatment of complexities associated with mTBI and post-deployment adjustment difficulties. Those who enroll in the PREP must be available for therapies for several hours a day over a period of a few weeks. Due to the qualifications and rigorous nature of the PREP program, many of these individuals may have been unemployed when they enrolled into the program.

The majority of the participants in our sample reported being deployed with a smaller number noting two or more deployments. A large proportion of those deployed reported exposure to blasts, which is an area of research that has yet to be fully elucidated. Multiple complex relationships have been found between multiple deployments and blast exposures [40], [41]. There appears to be a strong correlation between exposure to a blast and an individual's physical, cognitive, and emotional/psychological functioning. Thus, those exposed to a blast may experience various problems that could affect their ability to obtain steady employment.

We also found that many service members and Veterans reported having completed some college coursework, but did not obtain a degree (53%). Many of these individuals had a future goal of returning to school/training (60%) and then entering into the paid workforce (65%). These findings substantiate previous research that military advocates, educators, and researchers are concerned about post-secondary education attainment, and that complex relationships exist between TBI, PTSD, and educational attainment [42], [43]. Although it was beyond the scope of this study, future research should investigate reasons why service members and Veterans may be less likely to complete their degrees.

In this study, we were able to find the driving status of all Veterans and service members in the EHR records we reviewed. In our sample, over half of our participants were able to drive. Previous research has identified access to reliable transportation to be the biggest environmental barrier experienced by persons with TBI [44]. Self-reported driving problems are common with OIF/OEF Veterans [56]. Further, those who do not resume driving are at risk for poor community reintegration including occupational reintegration [45], [46], [67]. It is possible that service members and Veterans in our sample had access to reliable transportation, but adjustment to driving in civilian environments or other comorbid factors associated with TBI [56] may contribute to the high unemployment rates found in this study.

Finally, we found that approximately 19 (32%) of the participants in this study had CareerScope [47] assessment profiles which include interest inventory clusters. The interest inventory measures and identifies careers of interest that correspond to the US Department of Labor's interest areas. Of the twelve broad interest areas, the most frequently reported interest areas were 04-Protective, 02-Scientific, 05-Mechanical, 11-Leading/Influencing, 12-Physical/Performing, and 07-Business Detail. These findings were not surprising since these areas of interest mirror many of the Military Occupational Specialties (MOS) reported in the EHR. Research has shown that a number of factors have been associated with successful return to employment [35], [65] including premorbid employment status [16], [17], [66]. Further, the nature of pre-injury employment has been recognized as a significant pre-injury factor [57].

These findings may indicate that vocational rehabilitation counselors and other rehabilitation service providers need to heighten their awareness of the specific work histories, career interests, and needs of service members and veterans with TBI entering or reentering the civilian workforce or post-secondary education [48]–[50].

### Limitations of the Study

Limitations of this pilot study include a small sample size consisting of mostly Caucasian male service members and Veterans who all participated in the PREP program. How these findings generalize to other racial groups and females is unknown. Additionally, because the PREP program is in-patient, many of these service members and Veterans may have been unemployed for extended periods. However, many service members and Veterans who experience mTBI may struggle with unemployment. Additionally, the present study utilized records from a large VA hospital indicating that results may be more representative of the Veteran population due to the high volume of clients at this facility.

Due to the design of this study, it is difficult to determine whether each barrier identified in this study may be a cause, effect, or incidental correlate of employment status. As stated previously, the PREP program is intensive and many of the individuals are unemployed. Perhaps if researchers are able to determine specific declines in functioning following deployment, it can be linked to the injuries they may have received such as mTBI. It is possible that this would allow for a greater proportion of needs to be met and a higher likelihood of return to work for veterans with mTBI. An additional limitation is that only a single annotator performed the chart review. Future studies should include two annotators and an adjudicator to provide additional chart review reliability.

## Conclusions/Future Directions

Employment information on a unique sample of service members and Veterans who completed the TBI PREP in-patent rehabilitation program was reviewed in order to 1) develop an annotation schema and a training set of annotated documents to perform information extraction (IE) on text notes available in the VA EHR, and 2) to determine if information about employment status, goals and work-related challenges are identifiable in the EHR. In our study, we found that limited administrative (structured or coded) data in the EHR are available on the employment status of Veterans and service members with mTBI. Further we found that data about employment and factors that may affect return-to-work are identifiable and available in clinical text notes in the EHR.

Findings in our pilot study may improve our understanding of the myriad of employment challenges experienced by these individuals. Study findings add to our current knowledge and understanding about the diverse reintegration challenges experienced by service members and Veterans with mTBI. Overall, we found that service members and Veterans with mTBI experience high rates of unemployment, while many of these individuals have future goals of returning to school and/or paid employment. These findings suggest that service members and Veterans with mTBI experience a number of symptoms that may affect their ability to reintegrate into the workforce or post-secondary education. Concurrently addressing health-related, vocational, and independence needs may help alleviate gaps in treatment planning and service delivery to Veterans experiencing post-deployment stress and barriers to community reintegration [71]. It is important for clinical team members have access to current and accessible information on vocational status, goals, and driving ability. This practical information may be used to guide specialized evaluations and inform treatment planning to help facilitate reintegration.

Larger-scale retrospective and prospective studies are needed to advance our understanding of the unique reintegration needs of a population of service members and Veterans that is not fully elucidated. Natural Language Processing (NLP) may be a system to consider using to automatically extract great amounts of text-based employment information from a large sample, which will assist clinicians, vocational service providers, and researchers. Future research is needed to further our understanding of the reintegration needs and provide information on ways to best support Veterans as they enter or reenter the workforce [71]. Increased awareness of the challenges experienced by service members and Veterans with TBI may aid in an improved understanding of symptoms that are important to target for future interventions that may improve community re-integration outcomes.
